# Editorial: Neuro-covid: neuropsychological implications of the pandemic

**DOI:** 10.3389/fpsyg.2022.971780

**Published:** 2022-07-18

**Authors:** Martina Amanzio, Sara Palermo, George Prigatano, Irene Litvan

**Affiliations:** ^1^Department of Psychology, University of Turin, Turin, Italy; ^2^Neuroradiology Unit, Diagnostic and Technology Department, Fondazione Istituto di Ricovero e Cura a Carattere Scientifico (IRCCS), Istituto Neurologico Carlo Besta, Milan, Italy; ^3^Department of Clinical Neuropsychology, Barrow Neurological Institute, Phoenix, AZ, United States; ^4^Parkinson and Other Movement Disorders Center, University of California, San Diego, San Diego, CA, United States

**Keywords:** COVID-19, SARS-CoV-2, brain dysfunction, neurology, neuropsychology, cognitive dysfunctions, mood changes, pandemic fatigue

The increased understanding of the impact of COVID-19 on the nervous system has led to the increasing use of the term neuro-COVID (Chiappelli, 2020), which refers to the set of neurological and neuropsychological disorders seemingly caused by Coronavirus (SARS-CoV-2) infection that may affect the nervous system as a whole, including the brain and spinal cord.

The involvement of the Central and Peripheral Nervous Systems has already been documented in SARS patients, however SARS-CoV-2 appears more aggressive than other coronaviruses. The interaction between the human host and the virus can lead to neuronal dysfunction *via* an inflammatory mechanism (Chiappelli, 2020; Whittaker et al., 2020). Several studies show that many patients remain neurologically impaired even after recovery. These symptoms include confusional state, dizziness, headache, loss of smell and taste, dysphasia, and reduced visual acuity. Other complications include cerebrovascular accidents, and seizures. Peripheral nerve involvement has also been noted with polyneuropathies such as Guillain-Barré syndrome and Miller-Fisher syndrome (El Otmani and Moutaouakil, 2020; Whittaker et al., 2020). As a result of the infection, mood abnormalities, fatigue, and cognitive impairment were also reported (Bartoli et al., 2020) As a final note, the neurological, neuropsychological, and psychosocial impact of COVID-19 on patients with pre-existing brain disorders is particularly important in hospitalized and outpatient settings as well as home management (Palermo, 2020, 2021).

Throughout this Research Topic, we provide an innovative overview of the growing body of knowledge surrounding the multiple ways that COVID-19 impacts the human brain, causing long-term sequelae and their effects on cognitive function, behavior, and quality of life in affected individuals. We presented the research efforts aimed at understanding neuro-COVID and the role that neurology and neuropsychology can play in solving these challenges. A modern multidisciplinary perspective integrated with a variety of research methods to emphasize neuroanatomical circuits and mechanisms of neurochemical modulation in human subjects with COVID-19 is proposed.

To achieve this goal, the Authors who participated in this Research Topic would have to consider both patients with pre-existing brain disorders as well as normal subjects while examining the neuropsychological and psychosocial impact of the pandemic. A growing awareness of the benefits of highly specialized neurorehabilitation on patients with neuro-COVID can benefit from such an integrated approach.

This Research Topic comprises eight contributions consisting of four original research articles, two full reviews, one brief research report, and one case report. Overall, the scientific contributions published in this Research Topic exemplify the great complexity hidden behind the neurological and neuropsychological complications of COVID-19 infection.

Current evidence strongly suggests that patients surviving COVID-19 are at high risk for the subsequent development of neurological disease (Alipoor et al.). In their review, these investigators found that even when the clinical symptoms resolved, SARS-CoV-2 might persist within the Central Nervous System and cause chronic and latent infection in a large proportion of the population including those who suffered only mild respiratory symptoms. It will need to be determined whether this chronic infection could lead to future neurodegenerative disorders, particularly in those who are susceptible.

In support of this pathogenetic mechanism, Daroische et al. found that patients with recent SARS-CoV-2 infection appear to experience global cognitive impairment, memory, attention and executive and verbal fluency dysfunctions. Consequently, the authors recommend that clinicians consider a cognitive assessment of patients with recent COVID-19 infection, whatever disease severity, length of intensive care unit stay and type of interventions received.

Of all the possible interventions, amantadine was found to be associated with a reduced risk of COVID-19 infection. Kamel et al. proposed that the mechanism is inhibition of the E-channel conductance in reconstituted lipid bilayers of severe acute respiratory syndrome. These authors emphasized that contact with COVID patients and old age are risk factors for COVID-19 infection. This is in line with other research (Bartoli et al., 2021; Cipriani et al., 2021).

Indeed, the COVID-19 pandemia is a health issue leading older adults to an increased vulnerability to adverse outcomes. Amanzio et al. verified in a longitudinal study the role of neuropsychogeriatric factors associated with lockdown fatigue in healthy cognitive aging. They confirmed that cognitive, psychological, and physical factors play a complex interrelationship in the emergence of pandemic fatigue in the older population.

A case report on a 67-year-old woman with Alzheimer's disease, shows how cognitive frailty associated with infection is a harbinger of “brain fog” (Matias-Guiu et al.). These investigators substantiate what has emerged from previous works: as there are multi-domain complications, a comprehensive clinical, cognitive, and biomarker evaluation may disentangle the underlying COVID-19 mechanisms linked with cognitive dysfunction.

In line with the previous findings, original research on Brazilians confirmed that Parkinson's disease and Multiple sclerosis patients should be encouraged to perform more physical activity to reduce the effects of isolation and maintain motor and non-motor aspects of the diseases (Simieli et al.). They suggested that home-based exercise, should be included in the daily routine to reduce the impact of lockdown and maintain quality of life.

Data from another region of the world confirm the undeniable need to take charge of neuro-COVID, especially in frail subjects (Alkeridy et al.). Indeed, infections coupled with neurological manifestations in people living in Saudi Arabia, with the elderly and those with underlying neurological disorders have more risk.

All this leads to the importance of monitoring discharged patients. A brief research report took into consideration the data that emerged from a neurological outpatient clinic for patients with post-COVID-19 syndrome (Boesl et al.). The identification of clinical phenotypes and diagnostic subcategories is essential for improved patient care. Neurological sequelae could persist for more than 3 months after acute SARS-CoV-2 infections, even if mild.

The long-term neuropsychological consequences of Neuro-Covid remain to be determined. By way of example, the causes of temporary and extended neurocognitive impairment after severe COVID-19 disease have not yet been fully elucidated. On these bases, research is ongoing on biomarkers and imaging findings. The Karolinska NeuroCOVID study protocol (Nelson et al., 2022). Research focused on neurodegenerative and neuroinflammatory processes to better understand the pathology and neurological consequences of infection, and to identify therapeutic targets (Nelson et al., 2022). Furthermore, post COVID-19 individuals are at increased risk of chronic pain and dissecting the pathogenic (and clinical) aspects of this phenomenon is not easy (Cascella et al., 2021). The Research Topic needs more research and must be approached from a molecular perspective, seeking to provide explanations for the underlying pathophysiological processes (Cascella et al., 2021).

Some methodological problems concerning neuro-COVID research also remain. To date, many studies do not use adequate control groups. We therefore urge caution about the inference that the long term poor neuropsychological test performance is clearly connected to permanent brain changes caused by COVID infection. Nevertheless, the picture that has emerged from the research presented here agrees that the elderly population may be especially vulnerable, but more research needs to be conducted in this area with appropriate controls. Future research will also allow to quantify the risk of developing specific neurological disorders in patients with COVID-19 infection and the strength of their causal relationship, while assessing the risk of chronicity of these disorders.

## Author contributions

All authors listed have made a substantial, direct, and intellectual contribution to the work and approved it for publication.

## Conflict of interest

The authors declare that the research was conducted in the absence of any commercial or financial relationships that could be construed as a potential conflict of interest.

## Publisher's note

All claims expressed in this article are solely those of the authors and do not necessarily represent those of their affiliated organizations, or those of the publisher, the editors and the reviewers. Any product that may be evaluated in this article, or claim that may be made by its manufacturer, is not guaranteed or endorsed by the publisher.
